# Retinoic Acid Alleviates TGEV-Induced Ferroptosis by Activating the p62-NRF2-GPX4/HO-1 Pathway and Iron Metabolism in Intestinal Epithelial Cells

**DOI:** 10.3390/nu18060994

**Published:** 2026-03-20

**Authors:** Conghui Yin, Xin Lai, Junning Pu, Chen Liu, Yuheng Luo, Jun He, Bing Yu, Lianqiang Che, Quyuan Wang, Huifen Wang, Daiwen Chen, Aimin Wu

**Affiliations:** 1 Institute of Animal Nutrition, Sichuan Agricultural University, Chengdu 611130, China; 18735193091@163.com (C.Y.); xin.lai2018@gmail.com (X.L.); junningpu@163.com (J.P.); liuchen@sicau.edu.cn (C.L.); luoluo212@126.com (Y.L.); hejun8067@163.com (J.H.); ybingtian@163.com (B.Y.); che.lianqiang@sicau.edu.cn (L.C.); wangqy@sicau.edu.cn (Q.W.); wanghuifen1005@163.com (H.W.); 2 Key Laboratory for Animal Disease-Resistance Nutrition of China Ministry of Education, Sichuan Agricultural University, Chengdu 611130, China

**Keywords:** transmissible gastroenteritis virus (TGEV), ferroptosis, reactive oxygen species (ROS), retinoic acid (RA), p62-NRF2-GPX4/HO-1 signaling pathway

## Abstract

**Background**: Transmissible gastroenteritis virus (TGEV) is a highly pathogenic porcine coronavirus that causes severe gastrointestinal damage in piglets. However, how TGEV affects host iron homeostasis, oxidative stress, and the ferroptosis process remains unclear. This study aimed to investigate the effects of TGEV infection on cellular iron metabolism, oxidative damage, and lipid peroxidation-mediated ferroptosis, as well as to evaluate the potential therapeutic role of retinoic acid (RA). **Methods**: Using an intestinal epithelial cell model of TGEV infection, we assessed key regulators of iron handling, oxidative stress, lipid peroxidation, and ferroptosis. The expression of ferroportin (FPN) and ferritin (FTH/L) and the activity of the p62–NRF2–GPX4/HO-1 antioxidant axis were analyzed, and the effects of exogenous RA treatment on these endpoints were examined. **Results**: TGEV infection disrupted cellular iron homeostasis by downregulating the expression of ferroportin (FPN) and ferritin (FTH/L), leading to the accumulation of intracellular free iron, which in turn induced the generation of a large amount of reactive oxygen species (ROS) and ultimately triggered ferroptosis in intestinal epithelial cells. Additionally, TGEV infection significantly inhibited the p62-NRF2-GPX4/HO-1 antioxidant signaling pathway, further exacerbating the ferroptosis process. **Conclusions**: This study reveals that ferroptosis is a key pathological mechanism in TGEV-induced intestinal injury and demonstrates that RA exerts a therapeutic effect by regulating iron metabolism and activating the p62-NRF2-GPX4/HO-1 signaling pathway. These findings provide new theoretical insights for potential intervention strategies targeting virus infection-associated ferroptosis and intestinal damage.

## 1. Introduction

TGEV is classified as a Coronaviridae virus with an envelope and a positive-sense single-stranded RNA genome [1]. TGEV is a major etiological agent of diarrheal disease and enteritis in swine. It affects pigs across all age groups with high morbidity, and the outcome is particularly severe in suckling piglets, in which mortality can approach 100% among those younger than two weeks [2]. Following TGEV infection, villous atrophy typically develops within 48 h and is subsequently accompanied by crypt hyperplasia. This pathological progression is associated with profuse watery diarrhea, which can rapidly cause severe dehydration and death in piglets [3]. TGEV induces intestinal barrier injury and leads to dysregulated intestinal homeostasis [2,4]. TGEV occurs worldwide and imposes substantial economic burdens on swine/pork production [5,6]. Thus, elucidating the mechanisms underlying TGEV pathogenesis and developing effective treatments remains a major priority.

Accumulating evidence indicates that viral infections can elicit non-apoptotic programmed cell-death pathways, with ferroptosis being one representative modality [7,8], a form of regulated cell death in which iron fuels lethal lipid peroxidation [9]. This process is orchestrated by regulators linked to amino acid handling, iron homeostasis, lipid turnover, and mitochondrial function [10], such as glutathione peroxidase 4 (GPX4) [11], ferroptosis suppressor protein 1 (FSP1) [12,13], and nuclear factor erythroid 2-related factor 2 (NRF2), etc. [14]. Within this regulatory network, NRF2 exerts an anti-ferroptotic effect by restraining intracellular ROS accumulation and reducing iron import. Conversely, several pro-ferroptotic factors facilitate this process: NADPH oxidase boosts ROS production, transferrin receptor 1 (TFR1) enhances cellular iron uptake, and p53 contributes by suppressing SLC7A11 expression [15]. Accumulating evidence links ferroptosis to a broad spectrum of pathological conditions, such as neurodegenerative disorders, cancer, tissue injury, and infections caused by pathogens [16,17]. Moreover, a growing body of evidence indicates that several viruses, including SARS-CoV-2, hepatitis B virus, and influenza A virus, are capable of triggering ferroptotic cell death in host cells. This effect has been attributed to disruptions such as iron accumulation, depletion of glutathione, and activation of ferritinophagy [8,18,19]. In SARS-CoV-2 infection, for example, ferroptosis contributes to pulmonary epithelial injury by promoting lipid ROS generation and inhibiting antioxidant defense systems [18]. Notably, oxidative stress and mitochondrial damage—both hallmarks of TGEV infection [1]—are recognized as key upstream events in ferroptosis. Accordingly, we propose that TGEV infection could promote ferroptotic cell death in intestinal epithelial cells through the engagement of iron-associated signaling, thereby exacerbating intestinal injury.

Retinoic acid (RA) is widely recognized as a key bioactive molecule involved in embryogenesis, cellular differentiation, immune modulation, and therapeutic regulation in disease contexts. By counter-regulating the expression of hepcidin and transferrin (Tf), RA rectifies the LPS-induced imbalance of iron metabolism in acute inflammation [20]. Collectively, these data imply that RA may function as a suppressor of ferroptotic cell death. Although RA has been extensively investigated in recent years, its involvement in ferroptosis, particularly in the context of TGEV infection, remains largely unexplored, and mechanistic evidence is still limited.

This study examines how ferroptosis contributes to TGEV infection and replication, and the mechanism by which RA counteracts the virus by inhibiting ferroptosis. Collectively, our findings indicate that TGEV infection promotes ferroptotic cell death in intestinal epithelial cells through suppression of the p62-NRF2–GPX4/HO-1 signaling axis. Importantly, RA protects against TGEV infection by modulating ferroptosis and enhancing the expression of intestinal tight junction proteins, highlighting the therapeutic potential of targeting ferroptosis with RA.

## 2. Materials and Methods

### 2.1. Study Design

To investigate the mechanism of TGEV-induced ferroptosis and the protective effects of RA, this study employed both in vitro and in vivo models. For the in vitro experiments, IPEC-J2 cells were randomly assigned to different groups: (1) Control group (mock-infected); (2) TGEV group (infected with TGEV at an MOI of 1 for 36 h); and (3) TGEV + RA groups (treated with varying concentrations of RA [25, 50, 75, 100 μM] following TGEV adsorption). All in vitro cellular assays, including viability, flow cytometry, and protein expression analyses, were performed with a minimum of three independent biological replicates (*n* ≥ 3).

For the in vivo experiments, a total of 32 healthy crossbred weaned piglets (Duroc × Landrace × Yorkshire, aged 21 days) were randomly allocated into four experimental groups (*n* = 8 per group): (1) Control; (2) TGEV-challenged; (3) TGEV + RA-5 (treated with 5 mg/kg RA); and (4) TGEV + RA-15 (treated with 15 mg/kg RA). Following a 3-day acclimation period, piglets in the RA treatment groups were orally administered RA for 3 consecutive weeks. Subsequently, piglets in the TGEV-challenged and RA-treated groups were orally challenged with a single dose of TGEV (2.8 × 10^9^ PFU). At 3 days post-infection, which corresponds to the peak of diarrhea, all piglets were humanely euthanized. Blood samples were collected from the anterior vena cava to obtain serum. For histological analysis, jejunal tissues were fixed in 4% paraformaldehyde, routinely dehydrated through a graded ethanol series, cleared in xylene, and embedded in paraffin. Paraffin-embedded tissues were sectioned at 4–5 μm thickness, mounted on glass slides, deparaffinized, and rehydrated. The sections were then stained with hematoxylin and eosin (H&E), dehydrated again, cleared, and sealed for microscopic examination. Histological images were captured under a light microscope and used for subsequent morphological evaluation. Histological sections were examined and imaged using light microscope (Olympus BX43, Olympus Corporation, Tokyo, Japan) under bright-field conditions. Representative fields were captured at the same magnification and under consistent imaging settings across all groups to ensure the comparability of morphological evaluation. For morphometric quantification, at least 10 intact, well-oriented villi and their associated crypts per section were randomly selected. Villus height (defined as the distance from the villus tip to the villus–crypt junction) and crypt depth (defined as the distance from the villus–crypt junction to the crypt base) were precisely measured using Image J 1.53c at 40× magnification.

### 2.2. Measurement of Serum Iron Indicators

Serum iron, unsaturated iron binding capacity (UIBC), total iron binding capacity (TIBC) and transferrin saturation (TF%) were analyzed by Iron/TIBC Reagent Kit (I750460, Pointe Scientific, Canton, MI, USA).

### 2.3. Cell Culture

IPEC-J2, a porcine intestinal epithelial cell line, was cultured in DMEM/F-12 medium (Gibco, Waltham, MA, USA) containing 10% fetal bovine serum, 1% penicillin–streptomycin, 5 ng/mL human epidermal growth factor, and 20 μM HEPES. Cells were incubated at 37 °C under 5% CO_2_ in a humidified atmosphere and subcultured when they reached ~80% confluence.

### 2.4. Virus Infection and Retinoic Acid Treatment

The transmissible gastroenteritis virus (TGEV) strain used in this study was kindly provided by Prof. Zhiwen Xu. For infection experiments, IPEC-J2 cells were seeded into appropriate plates and allowed to grow to approximately 70% confluence. They were then infected with TGEV at an MOI of 1. Adsorption of the virus was performed at 37 °C for 60 min. The inoculum was then aspirated, and the monolayers were gently rinsed with PBS to remove non-adsorbed viral particles. Thereafter, fresh complete medium was added to continue the infection.

All-trans retinoic acid (RA) stock solutions were prepared in DMSO and freshly diluted with culture medium just prior to treatment. For post-infection intervention experiments, RA was added to the culture medium following viral adsorption and maintained for the indicated time periods. Control groups received an equivalent volume of DMSO.

### 2.5. Transmission Electron Microscopy (TEM)

For transmission electron microscopy, IPEC-J2 cells were collected and immersion-fixed in 2.5% glutaraldehyde at 4 °C overnight. The specimens were then treated with 1% osmium tetroxide for 2 h for postfixation and contrasted with 0.5% uranyl acetate. After dehydration in a graded ethanol series, samples were cleared with propylene oxide and embedded in epoxy resin. Using an ultramicrotome, ultrathin sections of approximately 80 nm were obtained. These sections were then observed under a transmission electron microscope (Tecnai 10, FEI, Hillsboro, OR, USA). Representative images were captured for morphological evaluation.

### 2.6. Western Blotting Analysis

Cells or intestinal tissues were homogenized in chilled lysis buffer containing a protease/phosphatase inhibitor cocktail (Thermo Fisher Scientific, Waltham, MA, USA). Protein levels were quantified using a BCA protein quantification kit. Protein samples of equal loading were resolved by SDS–PAGE and electrotransferred to PVDF membranes. Membranes were blocked with 5% skim milk and then incubated at 4 °C overnight with primary antibodies against markers of iron metabolism, oxidative stress, ferroptosis, and intestinal barrier integrity. For Western blot analysis, the following primary antibodies were used: p-NRF2 (Abcam, Cambridge, UK, ab76026), NRF2 (Proteintech, Rosemont, IL, USA, 16396-1-AP), FPN (Invitrogen, Waltham, MA, USA, PA5-22993), p62 (Abcam, ab56416), HO-1 (Proteintech, 10701-1-AP), GPX4 (Abcam, ab125066), FTL/H (Abcam, ab75973), ZO-1 (Invitrogen, UH289284), SI (Santa Cruz, Dallas, TX, USA, sc-27603), Occludin (Invitrogen, UA280516), Claudin 1 (Invitrogen, UB280983), and β-actin (Cell Signaling Technology, Danvers, MA, USA, 3700). All primary antibodies were diluted at 1:1000. HRP-conjugated goat anti-rabbit and goat anti-mouse secondary antibodies (Santa Cruz, sc-2030 and sc-2031) were used at a dilution of 1:3000. After incubation with HRP-linked secondary antibodies, the blots were developed with enhanced chemiluminescence (ECL) reagents. Protein loading was normalized to β-Actin.

### 2.7. Flow Cytometry Analysis of TGEV Infection

To detect TGEV-infected cells, cells were collected and washed once with PBS, followed by fixation in 4% paraformaldehyde for 15 min at room temperature. After centrifugation (500× *g*, 5 min), cells were permeabilized with 0.05% Triton X-100 (Sigma-Aldrich, Inc., St. Louis, MO, USA) for 10 min and incubated with a mouse monoclonal antibody against the TGEV nucleocapsid protein (Santa Cruz, sc-52436; 1:100, diluted in 0.05% Triton X-100/PBS) for 2 h at room temperature. After washing, cells were incubated with a FITC-conjugated goat anti-mouse IgG secondary antibody (Santa Cruz, sc-2010; 1:1000 in PBS) for 1 h in the dark. Finally, cells were washed again, resuspended in 500 μL PBS, and analyzed using a BD-FACS flow cytometer (Becton Dickinson, Franklin Lakes, NJ, USA). Flow cytometric data were processed using FlowJo 10.10.0 software.

### 2.8. Immunofluorescence Staining for TGEV Infection

After the indicated treatments, cells were washed with PBS, fixed with 4% pa. After the indicated infection and treatment procedures, IPEC-J2 cells were washed with PBS, fixed with 4% paraformaldehyde for 15 min at room temperature, and permeabilized with 0.05% Triton X-100 for 10 min. The cells were then incubated with a mouse monoclonal antibody against the TGEV nucleocapsid protein (Santa Cruz Biotechnology, sc-52436; 1:100, diluted in 0.05% Triton X-100/PBS) for 2 h at room temperature. After washing, the cells were incubated with a FITC-conjugated goat anti-mouse IgG secondary antibody (Santa Cruz Biotechnology, sc-2010; 1:1000) for 1 h in the dark. Nuclei were counterstained with DAPI, and fluorescence images were captured under identical exposure settings using a fluorescence microscope (Olympus, Tokyo, Japan, CKX53). Green fluorescence represented viral antigen, whereas blue fluorescence indicated nuclei.

### 2.9. Cell Viability Assay

Cell viability was determined by a CCK-8 colorimetric assay. IPEC-J2 cells were plated in 96-well plates (5 × 10^3^ cells/well) and exposed to the indicated treatments. At the specified time points, CCK-8 solution was added to each well and allowed to react at 37 °C for 1–2 h. The absorbance at 450 nm was then recorded using a microplate reader.

### 2.10. Measurement of Intracellular ROS and Lipid Peroxidation

Cellular reactive oxygen species (ROS) production was assessed with 2′,7′-dichlorodihydrofluorescein diacetate (H_2_DCFDA). For intracellular ROS detection, cells were loaded with 50 μM H_2_DCFDA and incubated at 37 °C for 30 min in the dark. Lipid peroxidation was evaluated by staining cells with the fluorescent probe C11-BODIPY (5 μM) for 30 min, followed by PBS washing. Fluorescence signals were acquired by flow cytometry, and mean fluorescence intensity (MFI) was used to quantify ROS generation and lipid peroxidation.

### 2.11. Determination of the Labile Iron Pool (LIP)

The intracellular labile iron pool (LIP) was assessed using the calcein-AM quenching approach. Cells were stained with 0.05 μM calcein-AM at 37 °C for 30 min and rinsed with PBS, followed by incubation in the presence or absence of deferoxamine (DFO, 100 μM) for 1 h. Fluorescence signals were collected by flow cytometry, and LIP was estimated from the DFO-induced increase in calcein fluorescence (i.e., the difference between DFO-treated and untreated samples).

### 2.12. Measurement of Glutathione (GSH) and Malondialdehyde (MDA)

Intracellular antioxidant status was assessed by quantifying GSH with a commercial kit (Beyotime Biotechnology, Shanghai, China), and lipid peroxidation was estimated by determining MDA using the corresponding assay kit (Beyotime Biotechnology, Shanghai, China). Absorbance readings were obtained on a plate reader and expressed relative to protein content.

### 2.13. Statistical Analysis

All assays included a minimum of three independent replicates, and values are reported as mean ± standard error of the mean (SEM). GraphPad Prism 9.5.1 was used for all statistical computations. An unpaired two-tailed Student’s *t*-test was used to assess statistical significance between two groups. Differences among multiple groups were assessed using one-way analysis of variance (ANOVA). A threshold of *p*-value < 0.05 was used to indicate statistical significance.

## 3. Results

### 3.1. TGEV Infection Induces Ferroptosis in IPEC-J2 Cells

**Figure 1:**
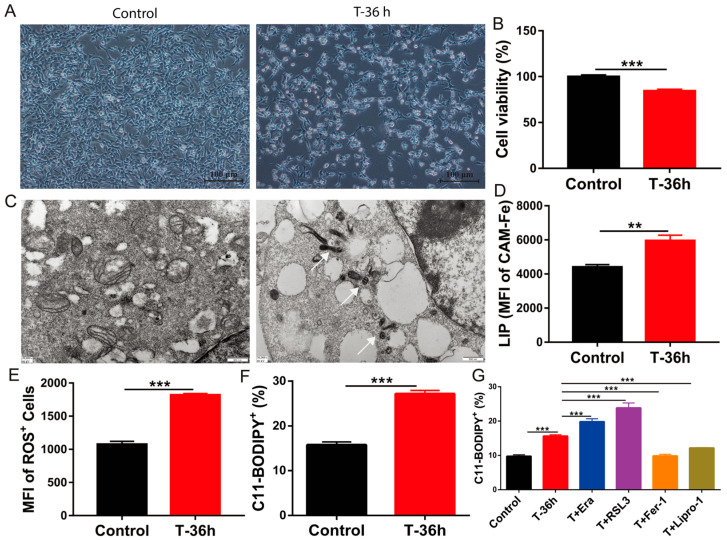
TGEV infection induces ferroptosis in IPEC-J2 cells. (**A**) Microscopic images showing morphological changes between the control and the TGEV-infected group (36 h post-infection). (**B**) Statistical analysis of cell viability between the control and the TGEV-infected group. (**C**) TEM images revealing ultrastructural alterations of mitochondria and cytoplasm between the control and the TGEV-infected group (scale bar: 500 nm). White arrows indicate morphologically damaged mitochondria in TGEV-infected cells. (**D**) Flow cytometry analysis of intracellular LIP between the control and the TGEV-infected group. (**E**) Fluorescence intensity analysis of intracellular ROS levels between the control and the TGEV-infected group. (**F**) Proportion of lipid peroxidation-positive cells (C11-BODIPY) between the control and the TGEV-infected group. (**G**) Effects of ferroptosis inducers (Erastin, 2 μM; RSL3, 1 μM) and inhibitors (Fer-1, 1 μM; Lipro-1, 1 μM) on lipid peroxidation in TGEV-infected IPEC-J2 cells. Data are presented as mean ± SEM of four independent biological replicates (*n* = 4). ** indicates *p* < 0.01, *** indicates *p* < 0.001.

TGEV infection caused evident cytotoxicity in IPEC-J2 cells, manifested by fewer cells and significantly reduced viability at 36 h post-infection (Figure 1A,B). Microscopic observations showed that cells infected with TGEV exhibited marked shrinkage, detachment, and cell damage compared to controls (Figure 1A). Transmission electron microscopy (TEM) further revealed ultrastructural alterations, including ruptured mitochondrial membranes, disrupted cristae, and cytoplasmic vacuolization, consistent with typical ferroptotic features (Figure 1C). The data from measurements of intracellular free iron (LIP), ROS, and the ferroptosis marker C11-BODIPY in TGEV-infected IPEC-J2 cells further support this conclusion (Figure 1D–F). Compared with uninfected controls, IPEC-J2 cells displayed significantly higher LIP levels after TGEV infection (Figure 1D), which was followed by a marked promotion of ROS production (Figure 1E). This ultimately led to a notable rise in C11-BODIPY levels (Figure 1F), thereby activating ferroptosis. Further intervention experiments showed that ferroptosis inducers Erastin and RSL3 further aggravated lipid peroxidation, whereas ferroptosis inhibitors Fer-1 and Lipro-1 markedly decreased lipid peroxidation (Figure 1G). Notably, the applied concentrations of these agents are well-established to cause no baseline cytotoxicity in uninfected IPEC-J2 cells [21,22]. Overall, our data suggest that TGEV infection perturbs iron homeostasis and promotes ferroptotic cell death, which in turn results in pronounced epithelial injury.

### 3.2. RA Addition Alleviates TGEV-Induced IPEC-J2 Cell Damage by Suppressing Ferroptosis

**Figure 2:**
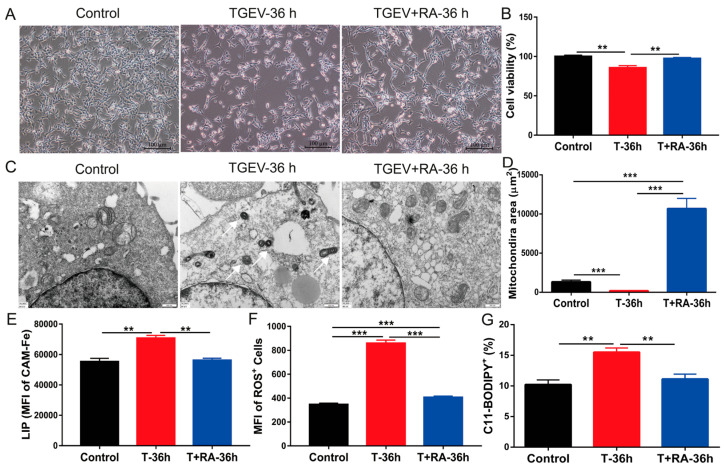
RA alleviates TGEV-induced IPEC-J2 cell damage by suppressing ferroptosis. (**A**) Microscopic observation of cell morphology in control, TGEV-infected (36 h), and RA-treated cells (scale bar: 100 μm). (**B**) Cell viability analysis of control, TGEV-infected (T-36 h), and RA-treated groups (T + RA-36 h). (**C**) TEM images depicting ultrastructural alterations of mitochondria and cytoplasm in each group (scale bar: 500 nm). White arrows indicate morphologically damaged mitochondria in TGEV-infected cells. (**D**) Statistical analysis of mitochondrial diameter in each group. (**E**) Flow cytometry fluorescence intensity analysis of intracellular LIP in each group. (**F**) Fluorescence intensity analysis of intracellular ROS levels in each group. (**G**) Analysis of lipid peroxidation levels indicated by the proportion of C11-BODIPY-positive cells in each group. Data are presented as mean ± SEM of four independent biological replicates (*n* = 4); ** indicates *p* < 0.01, *** indicates *p* < 0.001.

In the TGEV + RA-36 h group (treated with RA after TGEV infection), the cell state was improved compared with the TGEV-36 h group; the morphology was more regular, and the number of cells increased. RA administration alleviated the cell damage caused by TGEV infection to a certain extent (Figure 2A). TGEV challenge markedly compromised IPEC-J2 cell viability, whereas RA exposure restored cell survival (Figure 2B), indicating a protective effect of RA on cells. As observed by TEM, the mitochondrial structure in the TGEV + RA-36 h group was improved compared with that in the TGEV-36 h group, although there was still a certain degree of swelling and cristae damage; the membrane structure was relatively intact (Figure 2C). Quantitative analysis revealed that the mitochondrial diameter also increased significantly after RA treatment (Figure 2D). Notably, TGEV infection significantly increased intracellular LIP and ROS levels, whereas RA treatment effectively reduced their accumulation (Figure 2E,F). Similar results were also observed with C11-BODIPY (Figure 2G), indicating that RA effectively protects intestinal epithelial cells from TGEV-induced injury through inhibition of ferroptosis.

### 3.3. RA Addition Decreases TGEV-Induced Ferroptosis by Activating the p62-NRF2-GPX4/HO-1 Pathway and Iron Metabolism in IPEC-J2 Cells

**Figure 3:**
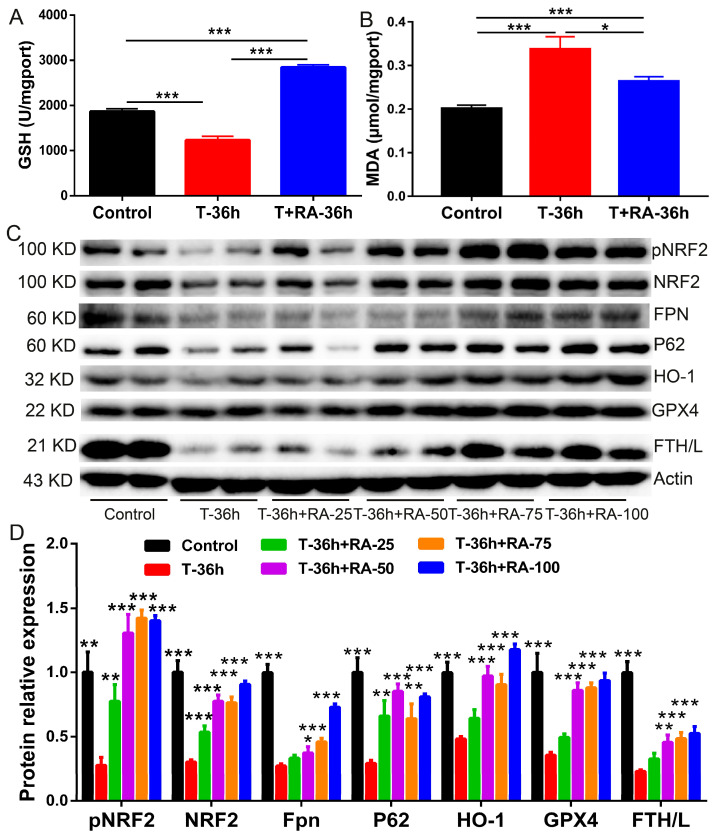
Retinoic acid restores redox homeostasis and activates NRF2-associated anti-ferroptotic responses in TGEV-challenged IPEC-J2 cells. (**A**) Intracellular GSH content. (**B**) MDA level as an index of lipid peroxidation. (**C**) Representative immunoblots of pNRF2, NRF2, FPN, p62, HO-1, GPX4, and FTH/L (β-actin as loading control) following T-36 h with increasing RA doses (25–100 μM). (**D**) Densitometric quantification of proteins shown in (**C**). For (**D**), all statistical comparisons are made versus the T-36 h (TGEV infection group). Data are presented as mean ± SEM of four independent biological replicates (*n* = 4). * *p* < 0.05, ** *p* < 0.01, *** *p* < 0.001.

To determine whether retinoic acid (RA) mitigates TGEV-driven oxidative injury and ferroptosis-related perturbations in intestinal epithelial cells, we quantified redox indices and profiled key regulators of iron handling and the p62–NRF2 antioxidant axis in IPEC-J2 cells. At 36 h post-infection, TGEV challenge significantly depleted intracellular glutathione (GSH) and increased malondialdehyde (MDA), indicating a pronounced shift toward oxidative stress and lipid peroxidation (Figure 3A,B). Notably, RA treatment robustly restored GSH and reduced MDA compared with the TGEV group, supporting an attenuation of lipid peroxidation burden (Figure 3A,B).

Mechanistically, immunoblotting revealed that TGEV exposure dampened NRF2 pathway activity and compromised ferroptosis defense, as reflected by reduced NRF2 phosphorylation and/or NRF2 abundance together with decreased expression of downstream cytoprotective proteins (HO-1) and the lipid peroxide detoxifying enzyme GPX4 (Figure 3C,D). In parallel, TGEV altered iron-metabolism-related markers (FPN and FTH/L) and lowered p62, consistent with impaired iron export/sequestration and a weakened p62-dependent antioxidant program (Figure 3C,D). Importantly, RA supplementation dose-dependently reactivated the p62–NRF2 axis, evidenced by increased pNRF2/NRF2 and upregulation of HO-1 and GPX4, accompanied by restoration of p62 and a corrective trend in iron-handling proteins (FPN and FTH/L) relative to TGEV alone (Figure 3C,D). Collectively, these results indicate that RA counteracts TGEV-induced oxidative stress and lipid peroxidation in intestinal epithelial cells, at least in part by re-engaging p62–NRF2–GPX4/HO-1 signaling and improving iron-homeostasis-associated protein expression.

### 3.4. RA Addition Inhibits TGEV Infection and Proliferation in IPEC-J2 Cells

**Figure 4:**
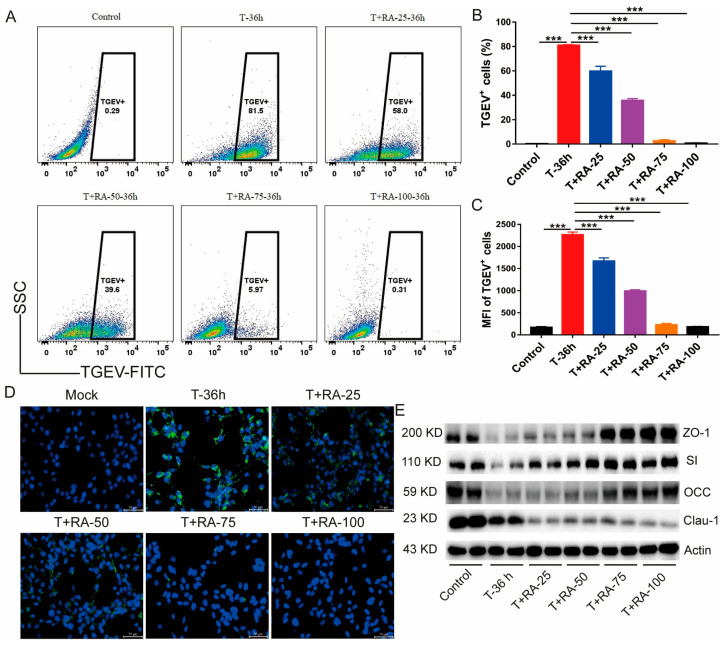
RA inhibits TGEV infection and proliferation in IPEC-J2 cells and improves intestinal barrier function. (**A**) Flow cytometry plots of TGEV infection in IPEC-J2 cells after RA treatment at various concentrations. (**B**) Statistical analysis of the percentage of TGEV-positive cells. (**C**) Statistical analysis of the mean fluorescence intensity (MFI) of TGEV-positive cells. (**D**) Immunofluorescence microscopy images of IPEC-J2 cells infected with TGEV under different treatment conditions (green: viral antigen, blue: nuclei). (**E**) Protein levels of ZO-1, occludin, claudin-1, and SI in IPEC-J2 cells under the indicated conditions were examined by Western blotting. Data are presented as mean ± SEM of four independent biological replicates (*n* = 4) for flow cytometry and 2 independent biological replicates (*n* = 2) for Western blot analysis. *** indicates *p* < 0.001.

Flow-cytometric quantification revealed that 81.5% of IPEC-J2 cells were TGEV-positive following infection. In contrast, RA markedly decreased the fraction of TGEV-positive cells. Increasing RA from 25 to 100 µM progressively enhanced its antiviral activity, with 100 µM RA nearly completely blocking viral infection and reducing the TGEV-positive rate to 0.31% (Figure 4A–C). These observations were corroborated by immunofluorescence staining, which showed an intense green signal in the TGEV-infected cells, indicating widespread viral infection, while in the RA-treated groups, particularly at 75 µM and 100 µM, the green fluorescence signal was significantly attenuated, demonstrating that RA effectively inhibits TGEV infection and replication (Figure 4D). WB showed that TGEV challenge markedly reduced ZO-1, occludin (OCC), and claudin-1 protein levels, along with SI expression. In contrast, RA treatment, especially at 100 µM, almost completely restored the expression of these proteins, indicating that RA can repair TGEV-induced intestinal barrier damage (Figure 4E). In summary, RA effectively inhibits TGEV infection and replication while protecting intestinal barrier function.

### 3.5. RA Addition Attenuates TGEV-Induced Intestinal Damage in Vivo

**Figure 5:**
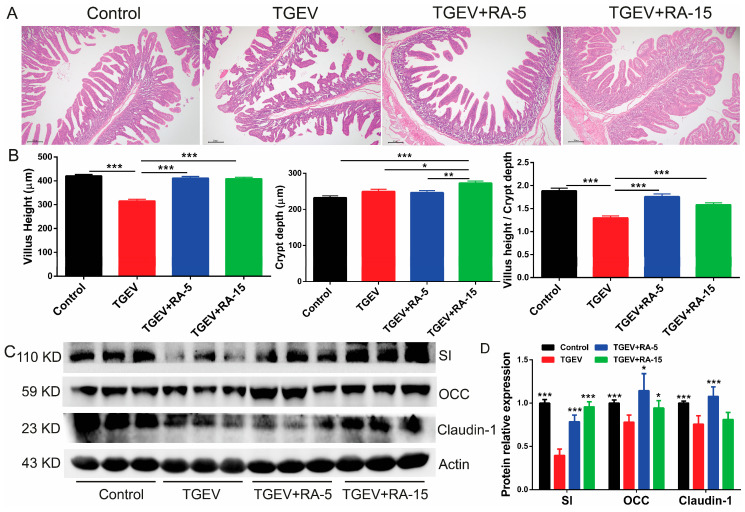
Retinoic acid significantly ameliorates intestinal barrier structural damage and restores tight junction protein expression in TGEV-infected piglets. (**A**) Representative HE staining images of small intestine sections from piglets under different treatment conditions (scale bar: 50 μm). (**B**) Statistical analyses of villus height, crypt depth, and villus height/crypt depth ratio of piglet small intestines among different groups. (**C**) Western blot analysis of tight junction proteins (OCC, Claudin-1) and SI expression in piglet small intestines under different treatment conditions. β-Actin was used as an internal control. (**D**) Band intensities of SI, OCC, and Claudin-1 were quantified by densitometry (normalized to a loading control) and presented as relative protein expression. All significance annotations indicate comparisons versus the TGEV group. Data are expressed as mean ± SEM of eighty independent biological replicates (*n* = 80) for villus/crypt analysis and three independent biological replicates (*n* = 3) for Western blot analysis; * indicates *p* < 0.05, ** indicates *p* < 0.01, *** indicates *p* < 0.001.

In our in vivo study, TGEV infection induced marked atrophy and structural disruption of small intestinal villi. RA treatment (TGEV + RA-5 and TGEV + RA-15 groups) significantly attenuated these injuries, with a pronounced restorative effect observed at the higher dose (15 mg/kg) (Figure 5A). Morphometric quantification showed that RA treatment markedly increased villus height and normalized the villus-to-crypt (V/C) ratio toward control values. (Figure 5B). Upon TGEV challenge, the levels of OCC, claudin-1, and SI were significantly diminished, as determined by Western blot analysis, whereas RA treatment, particularly in the TGEV + RA-15 group, markedly restored their expression to nearly normal levels (Figure 5C). Densitometric quantification (Figure 5D) of the immunoblots showed that, relative to the TGEV group, the Control group exhibited significantly higher protein levels of SI, OCC, and Claudin-1. RA partially reversed the TGEV-associated reductions: TGEV + RA-5 significantly increased SI, OCC, and Claudin-1, whereas TGEV + RA-15 significantly increased SI, with no significant changes in OCC or Claudin-1 (all comparisons vs. TGEV). Indicating a vital role of RA in mitigating TGEV-induced intestinal dysfunction. Together, these results indicate that RA protects against TGEV-induced intestinal structural and functional impairment in a dose-responsive fashion, with the strongest benefit observed at the higher dose (15 mg/kg), effectively restoring intestinal barrier integrity.

### 3.6. RA Addition Significantly Alleviates TGEV-Associated Oxidative Injury and Activates NRF2-Dependent Cytoprotective Responses In Vivo

**Figure 6:**
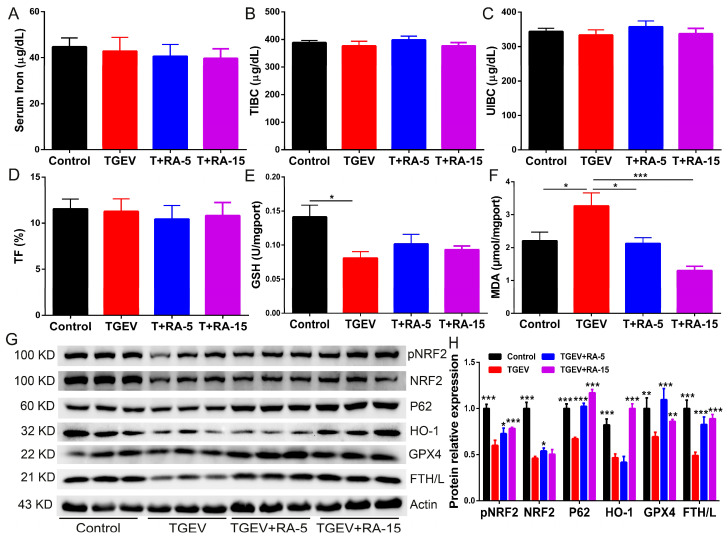
RA addition alleviates TGEV-associated oxidative injury and activates NRF2-dependent cytoprotective responses in vivo. (**A**–**D**) Serum iron indices, including serum iron (**A**), TIBC (**B**), UIBC (**C**), and transferrin saturation (TF) (**D**). (**E**,**F**) Redox status assessed by GSH content (**E**) and MDA level (**F**). (**G**) Representative immunoblots of pNRF2, NRF2, p62, HO-1, GPX4, and FTH/L (β-actin as loading control). (**H**) Densitometric quantification of proteins shown in (**G**). For (**H**), all statistical comparisons are made versus the TGEV group. Data are presented as mean ± SEM of eight independent biological replicates (*n* = 8) for serum iron and anti-oxidative indicators, and three independent biological replicates (*n* = 3) for Western blot analysis. * *p* < 0.05, ** *p* < 0.01, *** *p* < 0.001.

To extend the in vitro findings to an in vivo setting, we examined systemic iron indices, oxidative stress status, and NRF2-associated cytoprotective signaling in TGEV-challenged animals with or without RA supplementation. Circulating iron parameters, including serum iron, total iron-binding capacity (TIBC), unsaturated iron-binding capacity (UIBC), and transferrin saturation (TF), remained largely unchanged among groups (Figure 6A–D).

In contrast, TGEV challenge induced a clear redox imbalance, characterized by decreased GSH and elevated MDA, consistent with antioxidant depletion and enhanced lipid peroxidation in vivo (Figure 6E,F). RA supplementation mitigated these oxidative abnormalities, partially restoring GSH and markedly suppressing MDA accumulation compared with the TGEV group, indicative of alleviated lipid peroxidation burden (Figure 6E,F). At the molecular level, RA also reinforced NRF2-dependent defense programs in TGEV-exposed tissues, as evidenced by increased pNRF2/NRF2 and elevated expression of HO-1 and GPX4, together with upregulation of p62 and FTH/L relative to TGEV alone (Figure 6G,H).

## 4. Discussion

Ferroptosis represents a form of regulated cell death that is independent of caspase activation and closely linked to autophagic processes, driven by iron-dependent lipid peroxide overload reaching fatal thresholds [23]. In general, iron-dependent cell death is driven by three interrelated events: (1) an expansion of the intracellular labile (“free”) iron pool, which promotes Fenton chemistry and oxidative stress; (2) depletion of the antioxidant glutathione (GSH), weakening redox buffering capacity; and (3) excessive lipid peroxidation, leading to membrane damage and degeneration [24]. Interestingly, TGEV infection directly decreases *Fth* and *Ftl* mRNA expression. It also induces ferritin degradation by activating ferritinophagy. This leads to intracellular free iron accumulation, which subsequently triggers ROS production via the Fenton reaction and causes lipid peroxidation. To counteract ROS generated by an expanded labile iron pool, cells increase the uptake of cystine/cysteine to support glutathione (GSH) biosynthesis. This import is primarily mediated by the cystine/glutamate antiporter system Xc^−^. This system is a heterodimer comprising SLC7A11 (for cystine translocation) and SLC3A2 (for proper system assembly and activity) [25]. Although TGEV infection regulates Slc7a11 mRNA and protein expression, TGEV infection inhibits GSH synthesis because of the downregulation of SLC3A2 expression. Notably, under physiological conditions, elevated HO-1 levels contribute to alleviating redox stress [26]. However, persistent elevation of HO-1 expression may conversely promote ferroptosis [27]. HO-1 degrades heme, releasing ferrous iron (Fe^2+^) together with carbon monoxide and biliverdin. When intracellular Fe^2+^ becomes excessively accumulated, this iron overload can directly drive ferroptotic cell death [28].

Accumulating evidence from diverse viral infection models indicates that ferroptosis contributes substantially to virus-associated host cell death [10]. For example, Cheng et al. studied influenza virus infection in human lung epithelial cells (A549). They found that the virus induced ROS accumulation, downregulated GPX4, and activated ferroptosis. Furthermore, ferroptosis inhibitors effectively slowed viral replication [29]. Using a bovine viral diarrhea virus (BVDV) infection model, Li et al. reported that BVDV facilitates ferroptotic cell death by driving mitochondrial impairment and excessive lipid peroxidation. Notably, modulation of this ferroptosis-related cascade was able to mitigate cellular inflammation and injury, revealing how the virus activates the inflammatory pathway by means of cell death mechanisms [30]. Xia et al. employed a coronavirus infection model in mouse lungs. Their findings suggest that ACSL1-mediated ferroptosis not only promotes viral replication but also causes significant lung injury, and its inhibitor showed significant anti-infection effect in animals [31]. In contrast, this study extended the ferroptosis mechanism to the porcine TGEV infection model and filled the research gap in this field.

Our findings indicate that TGEV causes intestinal epithelial injury by perturbing iron metabolic balance and engaging the ferroptosis pathway. TGEV markedly reduced FPN and FTH/L expression. This suggests a blockage of iron efflux and subsequent accumulation of intracellular free iron. Consequently, this iron overload likely promotes ROS generation and lipid peroxidation via the Fenton reaction [32]. This process coincides with a reduction in GPX4 levels, which inhibits glutathione synthesis and peroxide clearance, further enhancing the ferroptosis phenotype [11]. The NRF2 signaling axis serves as a key brake on ferroptosis; once activated, it promotes ROS detoxification and strengthens cellular antioxidant defenses [14]. We found that TGEV infection led to a pronounced reduction in NRF2 expression, along with a marked decrease in the NRF2 target genes *HO-1* and *GPX4*, and downregulated its activator p62. These results suggest that viruses may increase cellular oxidative stress and susceptibility to ferroptosis by disrupting antioxidant pathways. It has been confirmed that the p62-NRF2-GPX4/HO-1 pathway plays a central role in viral infection and cellular antioxidant reaction [33]. For example, in hepatitis B virus (HBV) and SARS-CoV-2 infection models, the virus also disrupts this pathway to promote oxidative stress and viral replication [18].

Current research on RA has focused on cell differentiation and growth, and the polarization of immune cells. There are few studies on iron metabolism. Substantial evidence indicates that RA participates in the control of oxidative stress and cellular differentiation. In particular, Chang et al. reported RA attenuates oxidative stress and dampens inflammatory responses via the PI3K/Akt–NRF2 signaling axis [27]. According to Iturralde et al., RA increases intracellular ferritin (FTH/L) expression, thereby mimicking an iron-overload condition [34]. Its ability to target the NRF2 pathway makes it superior to traditional antioxidants or single-function inhibitors, and has the potential to be a multifunctional antiviral candidate [12,13,26]. This study systematically evaluated the protective effects of RA. RA treatment increased the expression of FPN and FTH/L, helped re-establish iron metabolic homeostasis, and markedly reduced intracellular labile iron and ROS accumulation. In parallel, RA strengthened NRF2-dependent antioxidant signaling, leading to restored GPX4 and HO-1 levels. Although HO-1 can potentially promote ferroptosis by releasing ferrous iron during heme degradation [35], it exerts a purely protective role in our model. Because RA simultaneously enhances iron sequestration and export, as evidenced by the decreased LIP, any HO-1-derived iron is safely managed. This coordinated regulation allows the antioxidant benefits of HO-1 to predominate, acting synergistically with GPX4 to effectively suppress TGEV-induced lipid peroxidation and ferroptosis.

Crucially, our in vivo evidence provides important conceptual insights into the scale of iron dysregulation during TGEV infection. Despite the profound ferroptotic damage and iron mishandling observed within the intestinal tissue, circulating systemic iron parameters, including serum iron, TIBC, UIBC and transferrin saturation, remained largely unchanged. This finding indicates that TGEV-induced iron dysregulation is highly localized. The subsequent ferroptotic cascade occurs predominantly at the intracellular level within the intestinal epithelium. It does not represent a failure of systemic iron metabolism. This distinction is conceptually significant for understanding the pathogenesis of acute enteric viral infections, as it suggests that specific cellular microenvironments can undergo severe redox and iron imbalances even when overall systemic iron availability appears intact [36]. Furthermore, the ability of RA to mitigate this localized damage underscores its targeted efficacy at the tissue level.

Consistent with this potent tissue-level efficacy, evaluating the dose–response relationship of RA in vitro provides crucial clues regarding its precise mechanism of action. Structurally and pharmacologically, RA acts primarily as a transcriptional regulator rather than a direct chemical iron chelator or a simple radical scavenger [37]. In our study, the upregulation of iron-handling proteins (FPN, FTH/L) and the p62-NRF2-GPX4/HO-1 antioxidant axis showed robust responses even at lower to intermediate RA doses (25–50 μM). Conversely, while viral load decreased steadily, the most profound anti-viral effect, nearly completely blocking TGEV infection, required higher doses (75–100 μM). This divergence in dose-responsiveness strongly suggests that RA’s primary and initial mode of action is the transcriptional promotion of host antioxidant and iron-handling networks. By restoring intracellular redox and iron homeostasis at lower doses, RA progressively turns the cellular microenvironment into an inhospitable landscape for viral replication. Ultimately, at higher concentrations (100 μM), this fortified intracellular defense culminates in a near-total viral blockade. Furthermore, our findings show that high-dose RA almost completely restores the expression of key tight junction proteins (ZO-1, occludin, and claudin-1). This indicates that RA’s potent anti-ferroptotic effect synergizes with physical reinforcement of the intestinal epithelial barrier, restricting both initial viral entry and subsequent cell-to-cell spread [38].

Despite our promising findings, several limitations remain. First, the link between RA and NRF2 is primarily associative. The pathway’s activation might partly reflect an improved cellular redox state. Targeted genetic or pharmacological suppression of NRF2 is needed to prove direct causality. Second, we used the immortalized IPEC-J2 cell line. This model lacks the full complexity of the intestinal microenvironment. Future research should incorporate primary porcine enteroids. Third, our animal study used a controlled laboratory setting. We currently lack clinical validation in commercial swine farms. These limitations clearly guide our future directions. Future experiments will use targeted genetic tools to map RA’s precise molecular targets. From a translational perspective, RA shows strong potential as a nutritional feed additive. It could effectively protect piglets against TGEV and alleviate virus-induced intestinal damage. Targeting host ferroptosis offers a promising, host-directed antiviral strategy. Ultimately, large-scale clinical field trials are required to validate the practical application of RA in commercial swine production.

## 5. Conclusions

In conclusion, TGEV infection induces ferroptosis by disrupting iron homeostasis, inhibiting NRF2 signaling and promoting lipid peroxidation, leading to intestinal cell damage. RA attenuated TGEV-induced cellular injury, which was associated with enhanced p62-NRF2-GPX4/HO-1 activity and re-establishment of iron metabolic homeostasis, enhancing antioxidant capacity, and inhibiting ferroptosis. Together, our data uncover an unrecognized mechanism through which TGEV triggers ferroptosis in intestinal cells and support retinoic acid as a promising intervention to mitigate virus-associated and oxidative injury. Despite these findings, several limitations remain. The precise manner in which TGEV modulates NRF2 signaling, as well as the detailed mechanism underlying the protective action of RA, has yet to be clarified. Subsequent work should delineate the molecular links connecting TGEV-induced ferroptosis with oxidative stress and related regulatory networks, thereby informing more targeted approaches to prevent and manage TGEV infection.

## Data Availability

This study was not registered in a publicly accessible protocol repository, which is common for preclinical animal research. The datasets generated and/or analyzed in this work are available from the corresponding author upon reasonable request.

## Abbreviations

p62	Sequestosome 1
NRF2	Nuclear factor erythroid 2–related factor 2
pNRF2	Phosphorylated NRF2
GPX4	Glutathione peroxidase 4
HO-1	Heme oxygenase 1
FPN	Ferroportin
FTH/L	Ferritin heavy/light chain
ROS	Reactive oxygen species
FSP1	Ferroptosis suppressor protein 1
TFR1	Transferrin receptor 1
p53	Tumor protein p53
SLC7A11	Solute carrier family 7 member 11
SLC3A2	Solute carrier family 3 member 2
LPS	Lipopolysaccharide
HJV	Hemojuvelin
TFR2	Transferrin receptor 2
IPEC-J2	Intestinal porcine epithelial cell line J2
GSH	Glutathione
MDA	Malondialdehyde
ZO-1	Zonula occludens-1
OCC	Occludin
Claudin-1	Claudin-1
SI	Sucrase-isomaltase
TIBC	Total iron-binding capacity
UIBC	Unsaturated iron-binding capacity
TF	Transferrin
IL-6	Interleukin-6
IL-1β	Interleukin-1 beta
TNF-α	Tumor necrosis factor-alpha
Keap1	Kelch-like ECH-associated protein 1
RBC	Red blood cell count
HGB	Hemoglobin
HCT	Hematocrit
MCV	Mean corpuscular volume
MCH	Mean corpuscular hemoglobin
MCHC	Mean corpuscular hemoglobin concentration
